# In vitro, ex vivo, and in vivo models for dental pulp regeneration

**DOI:** 10.1007/s10856-023-06718-2

**Published:** 2023-04-01

**Authors:** Sofia Silvia Piglionico, Coline Pons, Olivier Romieu, Frédéric Cuisinier, Bernard Levallois, Ivan Vladislavov Panayotov

**Affiliations:** 1 LBN, Univ. Montpellier, Montpellier, France; 2 Centro de Investigaciones Odontológicas, National University of Cuyo, Mendoza, Argentina

## Abstract

Based on the concept of tissue engineering (Cells—Scaffold—Bioactive molecules), regenerative endodontics appeared as a new notion for dental endodontic treatment. Its approaches aim to preserve dental pulp vitality (pulp capping) or to regenerate a vascularized pulp-like tissue inside necrotic root canals by cell homing. To improve the methods of tissue engineering for pulp regeneration, numerous studies using in vitro, ex vivo, and in vivo models have been performed. This review explores the evolution of laboratory models used in such studies and classifies them according to different criteria. It starts from the initial two–dimensional in vitro models that allowed characterization of stem cell behavior, through 3D culture matrices combined with dental tissue and finally arrives at the more challenging ex vivo and in vivo models. The travel which follows the elaboration of such models reveals the difficulty in establishing reproducible laboratory models for dental pulp regeneration. The development of well-established protocols and new laboratory ex vivo and in vivo models in the field of pulp regeneration would lead to consistent results, reduction of animal experimentation, and facilitation of the translation to clinical practice.

**Graphical Abstract:**

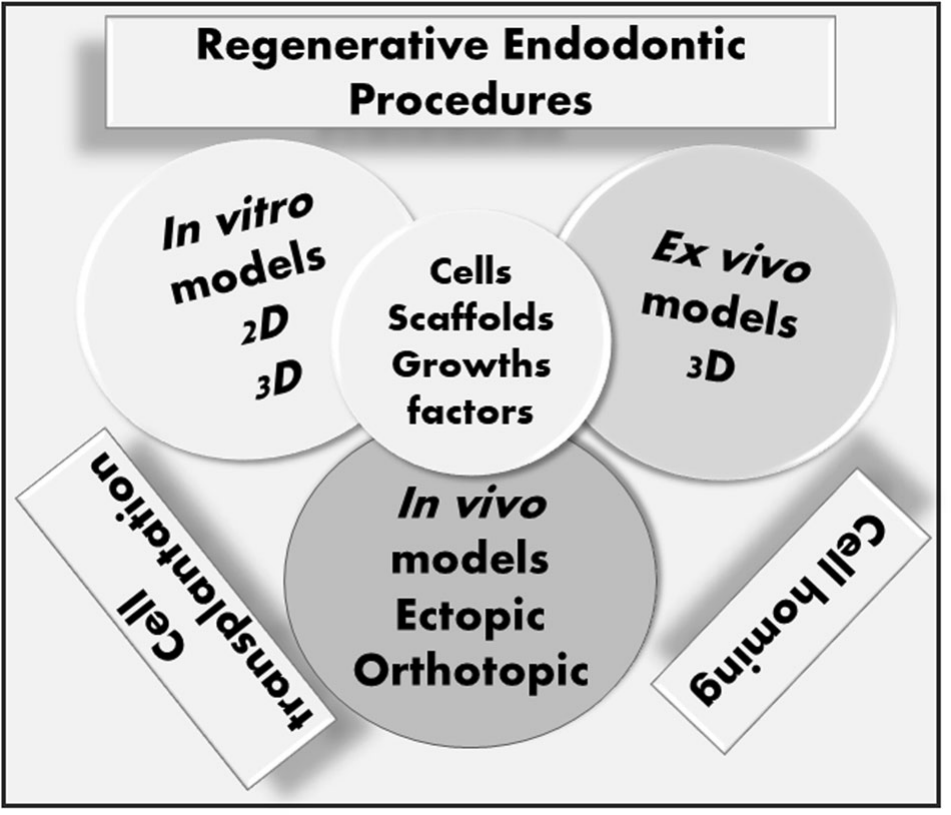

## Introduction

Following the Quality Clinical Guidelines for regenerative endodontic therapy published by the European Society of Endodontology (ESE) and by the American Association of Endodontists (AAE) (Table 1) [1] dental pulp regeneration approaches comprise two types of clinical procedures - Vital Pulp Therapies (VPT) that address vital teeth or Regenerative Endodontic Procedures (REP) of necrotic teeth. Pulp capping is a VPT applied when the healthy dental pulp has been minimally exposed. In this case, the direct application of a bioactive material would induce tissue response and regeneration preserving tooth vitality. The second regenerative procedure also known as “revascularization” is applied to necrotic teeth. It is a biological approach based on blood colonization of disinfected empty root canals by inducing periapical bleeding. The formed blood clot inside the canal acts as a scaffold containing growth factors from blood and dentin walls and undifferentiated cells from the periapical region which have migrated by “cell homing”.

**Table 1:** List of abbreviations

List of abbreviations	
AAE	American Association of Endodontists
ADSC	Adipose-Derived Stem Cells
BMDSC	Bone Marrow-Derived Stem Cells
BMP	Bone Morphogenetic Proteins
bFGF	Basic Fibroblast Growth Factor
CGF	Colony Growth Factor
DPSC	Dental Pulp Stem Cells
ECM	Extra Cellular Matrix
EDTA	Ethylene Diamine Tetra Acetic Acid
ESE	European Society of Endodontology
GF	Growth Factor
MSC	Mesenchymal Stem Cells
MTA	Mineral Trioxide Aggregate
PDLSC	Periodontal Ligament Stem Cells
REP	Regenerative Endodontic Procedures
SCAP	Stem Cells from the Apical Papilla
SHED	Stem Cells from Human-Exfoliated Deciduous Teeth
TGF	Transforming Growth Factor
VEGF	Vascular Endothelial Growth Factor
VPT	Vital Pulp Therapies

The beginning of pulp regeneration research was marked by the introduction of stem cells of dental origin [2]. The tool to describe these cells was classical 2D in vitro cell culture. However, these models did not simulate cell interaction with an extracellular matrix (ECM), so 3D in vitro models consisting of cell culture on a 3D matrix were introduced to evaluate cell-scaffold interactions [3, 4]. These models have been applied to test the effects of different scaffolds or bioactive molecules [5, 6]. Nevertheless, such in vitro models are far from reality and they do not guarantee adequate translation in clinical practice. The necessity of studies closer to real conditions led to the development of ingenious ex vivo models such as dentin-pulp slices [7], the entire tooth or crown culture [8, 9], and the mandible slice culture [10] to study pulp capping procedures, pulpitis [11, 12] or scaffolds for pulp regeneration [10].

The need to establish representative in vitro and ex vivo models for dental pulp regeneration resulted in the development of several in vivo experiments, making them the most widely spread models. Two categories of animal models were described: ectopic and orthotopic models. Ectopic models are farther from reality since they use Teflon tubes, dentin slices, or roots, containing a testing sample to be placed subcutaneously in the rodent’s back [13]. Orthotopic models are closer to clinical reality, the scaffolds and cells are placed in the pulp chambers or root canals of dogs [14]; porcine [15]; ferrets [16], or sheep [17].

**Fig. 1:**
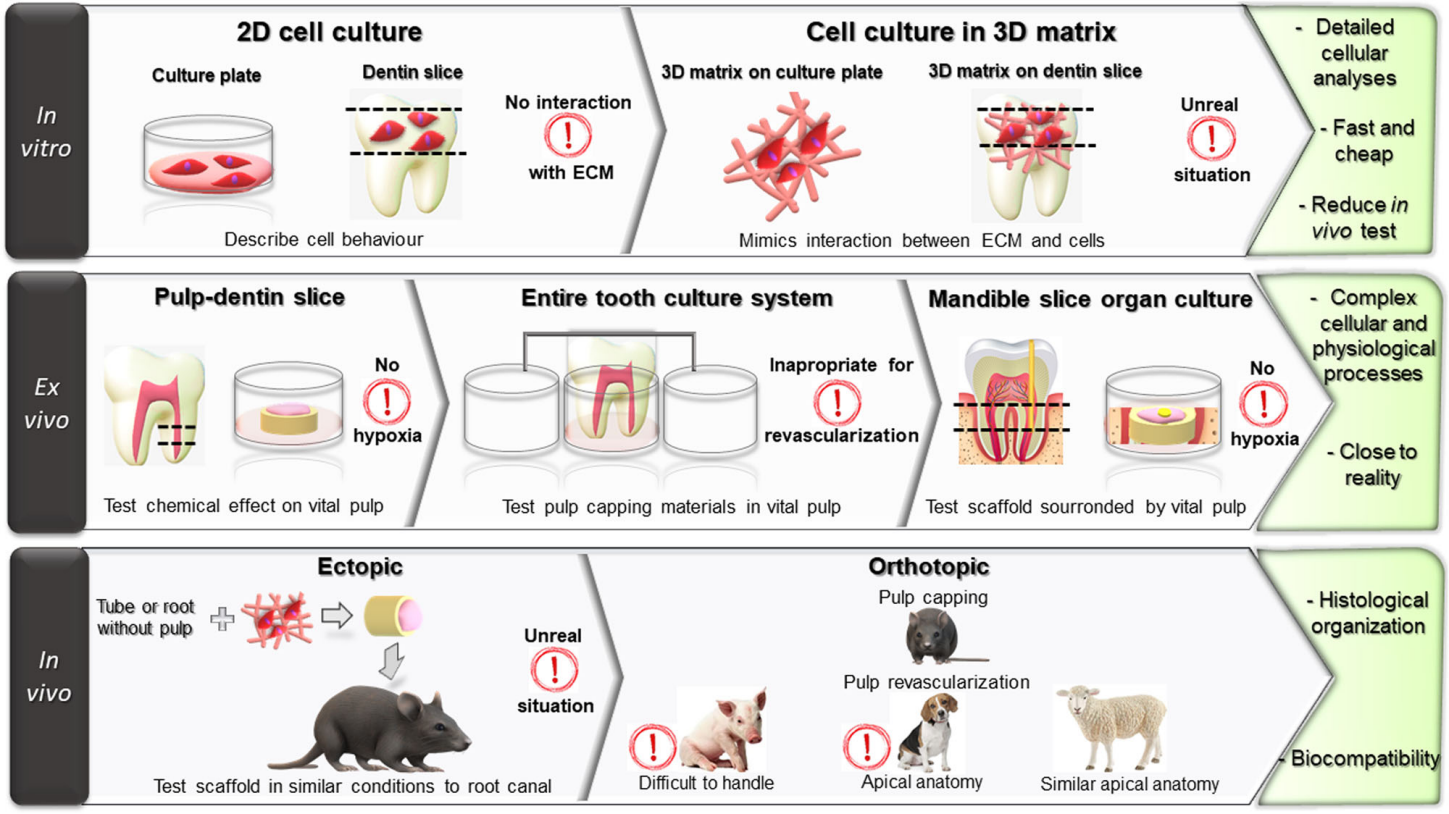
In vitro, ex vivo, and in vivo models developped for dental pulp regeneration

Hence a vast number of models for regenerative endodontics has been published and choosing one could be quite challenging when designing a study. Therefore with this narrative bibliographic review aimed to present and analyze in vitro, ex vivo, and in vivo models for dental pulp regeneration to help with the choice of a model (Fig. 1).

## Materials and methods

Analyzed articles were selected from Pubmed/Medline database following Problem, Intervention, Comparison, Outcome, Study (PICOS) criteria [18], where: P- regenerative endodontics, I- vital pulp capping for vital teeth, or regenerative endodontic procedure for necrotic teeth, C- comparison between study design using different cell lines, growth factors or scaffolds, O- dental origin cell behavior, pulp-like tissue formation, and S- in vitro, ex vivo, in vivo studies.

Exclusion criteria: randomized clinical trials, non-randomized clinical trials, prospective cohort studies, clinical cases, and articles whose methodology was inadequately explained.

## In vitro models for pulp regeneration

In vitro models used cells or biological molecules isolated from their natural environment to carry out experimentation outside a living organism in controlled laboratory conditions. They offer more detailed information about biological phenomena than studies in whole organisms. Besides, they are fast, cheap, and allow multiple experiments with a large number of samples thus reducing the number of in vivo studies/subjects needed [19]. However, extrapolation to humans is difficult and they lack data about biokinetics [20, 21].

Cell culturing in in vitro models can be performed in two-dimensional (2D) or tri-dimensional (3D) conditions. 2D models are used to describe cell behavior such as adhesion, proliferation, and differentiation and also to study the cytotoxicity of bioactive molecules. However, they are not adequate for studying tissue regeneration because they lack the interaction of tested components with the ECM.

To get over these disadvantages, 3D models have been developed; such as cellular spheroids, cell-laden hydrogels, mini-organs, and microfluidic organs-on-a-chip [22]. These 3D models better represent cell environments, allowing evaluation of the efficacy and safety of biochemical agents and modeling of biological processes [23]. A disadvantage of these models is the impossibility to mimic the interaction between ECM and different cell types [24]. In vitro models are summarized in Table 2.

**Table 2:** In vitro models for dental pulp regeneration

In vitro models						
Cells	Scaffold		Bioactive molecules			
			Chemotactic effect of GF after dentin conditioning		Biological effect of biomaterials for pulp capping	
2D	3D matrix	3D matrix + dental substrate	2D	3D	2D	3D
- Culture plate [25, 30–34] - Dentin surface [35]	-Hydrogels: *Classical* [38, 42–46] - Bio-printed [47, 48] - Pulp ECM [49–51]	- Hydrogel+dental root [54] - Hydrogel+dentin slices [38, 42, 53]	- Culture plate [55–57] - Dentin slices or powder [58–60] - Root Fragments [63] - Boyden chamber [61] - Boyden chamber with dentin [62]	- Hydrogel [49, 64] - Contidioned root canal + 3D matrix+ SCAP [65]	- Culture plate [66] - In vitro pulp chamber [67, 68]	- In vitro pulp chamber with 3D matrix [69] - Tooth-on-a-chip [70]

### In vitro models for cell behavior evaluation

Characterization of stem cells from dental origin has been achieved through 2D models. They allow different sources of cells involved in pulp regeneration processes to be described as cells from the dental pulp tissue (DPSC) [2, 25]; stem cells from the Apical Papilla (SCAP) of immature teeth, or from the Periodontal Ligament (PDLSC) [26, 27]. Mesenchymal Stem Cells (MSC) have been detected in the periapical zone so it is pertinent to consider them in pulp regeneration studies [28, 29].

All these cells have the potential to differentiate into odontoblasts, adipocytes, or neuron-like cells by culturing them in different media [30–32]. For odonto-osteogenic differentiation, basal media can be enriched with BMP-4, L-ascorbate-2-phosphate, dexamethasone, and phosphate [25]. Adipocyte-inducing medium is obtained by mixing insulin, isobutylmethylxanthine, and dexamethasone [33]. For neural differentiation, the neurobasal medium is supplemented with B27, epidermal growth factor, or fibroblast growth factor (FGF) [25, 34].

Later, 2D models conceived on dentin slices have been used to evaluate cell behavior on dentin surfaces. Dentin slices of human teeth were sterilized, placed on culture plates, and seeded with dental stem cells to evaluate adhesion, proliferation, and differentiation [35]. The advantage of this model is the use of dentin which represents a natural tooth tissue substrate and resembles clinical situations more than plastic plates. Moreover, it allows the evaluation of the effect of growth factors naturally trapped in the dentin collagen network. However, sterilization leads to the degradation of the bioactive factors. Other chemical treatments such as sodium hypochlorite or EDTA 17%, should be prioritized since they are considered to be less aggressive against bioactive molecules. Nevertheless, cell deposition in a liquid instead of in a 3D matrix on a dentin surface moves this model away from natural tooth conditions.

### In vitro models for scaffold evaluation

Accurate laboratory models are necessary to evaluate dental stem cell interactions in 3D matrices used as scaffolds for pulp regeneration since characteristics of cell environment such as surface chemistry, topography, and elastic modulus of the matrix influence local signals critical for cell behavior as survival, self-renewal, mobilization, proliferation and differentiation [36, 37].

#### 3D Matrices as scaffolds

3D matrices show beneficial effects on dental stem cell metabolism and differentiation because they provide a conducive environment in which dental repair can occur [38–40]. Hydrogels are the most recently adopted biomaterials as scaffolds in pulp regeneration since they are easy to apply, cheap and they have good mechanical and chemical properties [41]. The most widespread materials with beneficial properties for cell survival and proliferation are fibrin or collagen-based materials (Matrigel, Puramatrix) among others [38, 42–46]. These matrices are obtained by the polymerization of a solution containing cells or biomolecules that is injected inside the root canal where it jellifies. However, the polymerization process could be hazardous and entails dimensional changes.

To overcome these disadvantages innovative methods for hydrogel preparation as 3D bioprinting were applied. The technique relies on laser and cell-laden materials as ink to print fibers that could be placed inside the root canal to support the formation of pericyte microvascular networks [47]. Nonetheless, adaptation to root canal walls may not be good enough.

Out of the need to overcome this problem, a novel approach to bioprinting arose: The Light (Lithography) and Digital Light Processing (DLP) of a photo-crosslinked hydrogel that supports cell survival. The material is to be injected into the empty root canal and polymerized with a dental curing light [48]. It does not have the biochemical and biomechanical characteristics of the human dental pulp to induce cell metabolism and new tissue formation of a pulp-like tissue. That is why other types of materials have been proposed, such as the dental pulp decellularized ECM, alone or combined with biomaterials such as alginate [39, 49–51]. This scaffold supports the odontogenic differentiation of different cell types (BMSC, DPSC, and PDLSC) with no need of adding inductive biomolecules [52]. Results reflect the advantage of pulp ECM due to the presence of biochemical and biomechanical cues that render it a more physiologic environment for cells. However, the difficulty of this technique resides in the extirpation of a human dental pulp and shape adaptation to root canal walls.

#### 3D matrices on dental tissue substrate as a scaffold

To better mimic the dental pulp environment and assess the interaction between cells-scaffold and dental tissue, the complexity of the models increases. For instance, the use of dentin slices with a hydrogel containing cells was proposed to prove cell adhesion, survival, and proliferation that happen in contact with dentin [38, 44, 53].

An interesting approach is the full-length root canal model. It consists of constructed pre-vascularized root canals using a cell-laden hydrogel (gelatin methacryloyl) with encapsulated odontoblasts to obtain blood capillaries inside full-length root canals [54]. Human root fragments 9 mm long and with an apical diameter of 1.5 mm are sterilized, endodontically treated, rinsed with EDTA17%, and sectioned longitudinally in two parts to be re-attached. A prefabricated fiber is longitudinally positioned inside the canal and the hydrogel with cells is loaded into it and photopolymerized. Later, the central fiber is taken out, leaving a microchannel inside the hydrogel. Finally, endothelial cells are injected inside the channel resulting in a pre-vascularized full-length dental pulp-like tissue construct.

### In vitro models for bioactive molecules evaluation

Bioactive molecules are extensively applied in laboratory experiments due to their capacity to guide the healing process in pulp capping procedures and to induce cell homing in REP of necrotic teeth. They can be natural molecules as growth factors (GF), or chemical products present in biomaterials. To facilitate the description of in vitro models for pulp regeneration we classified biomolecules based on their application in two groups (Table 2).

- Evaluation of the chemotactic effect of natural biomolecules
- Evaluation of cytotoxicity and biocompatibility of biomolecules

### Chemotactic effect of natural biomolecules

Chemotactic effect of natural biomolecules as GF is necessary for cell-homing approaches, based on periapical cell migration inside the root canal space of a necrotic tooth. 2D in vitro cell cultures in plastic dishes have been used to analyze the influence of several GF (VEGF, BMP-2, FGF, TGF, CGF) in dental stem cell differentiation [55–57]. These studies allow the description of cell behaviors under the effect of biomolecules. However, they do not represent an environment close to reality since cells are cultured on a plastic substrate. The same procedures have been carried out in dentin slices or powder to prove the potential of dentin conditioning agents used in clinical procedures such as 17% EDTA, 10% citric acid, 1% phytic acid, or 37% phosphoric acid to release dentin GF and their beneficial effects regarding cell survival and proliferation [58–60].

Another 2D model used to assess the chemotactic effect of dentin GF is the Boyden chamber. It is a suspended hollow plastic chamber over a larger well. Both compartments are separated by a porous membrane. Cells are placed inside the upper chamber, and they could migrate through the membrane if the bioactive factors placed in the lower chamber have a cell mobilizing potential. One study used this chamber to assess liposomal delivery of encapsulated Demineralized Dentin Matrix, VEGF, and TGF-β1 proving that GFs from demineralized dentin matrix can recruit and promote odonto-differentiation of DPSC [61]. Further, the Boyden chamber was adapted to prove the release by EDTA of dentin chemotactic GF [62]. With this aim, EDTA pre-treated dentin discs were placed in the lower chamber and the migration of cells to non-treated and treated dentin was compared. Results proved that dentin released GFs trapped on the dentin matrix, enhancing cell migration.

Another study described an in vitro model consisting of root fragments treated by the conditioning agents mentioned above, to describe cell adhesion and morphology of adipose-derived MSCs attached to dentin [63]. The disadvantage of this model is the lack of a 3D matrix containing cells. A classical in vitro model with a culture plate with DPSC in a 3D matrix (Matrigel) has been performed to prove that VEGF and CGF enhanced cell proliferation, migration, and differentiation [49, 64]. Another study developed a more complex model based on the injection of a scaffold charged with cells inside root canal fragments pre-treated with different conditioning agents [65]. In this study, SCAPs were mixed with platelet rich-plasma and cultured into the root for 21 days. Results proved that irrigants such as EDTA 17% enhanced SCAP proliferation which could be beneficial in regenerative procedures. This model could be considered appropriate for studying the chemotactic effect of natural biomolecules since it represents a 3D matrix containing cells inside a natural root canal and it respects conditions such as hypoxia.

### Cytotoxicity and biocompatibility of biomolecules

To test cytotoxicity and biocompatibility of biomolecules and biomaterials the classical ISO cell test is applied [66]. It consists of the incubation of cells with biomolecules or biomaterials (cytotoxicity) or with a culture medium conditioned by the evaluated biomaterial (biocompatibility). This model is practical, cheap, and easy to develop giving clear results concerning cell biocompatibility.

More complex bi-dimensional devices have been developed, such as the in vitro pulp chamber model. This approach is useful to test the cytotoxicity of biomaterials based on the perfusion of molecules into dentinal tubes. This device is made of two chambers separated by a dentin slice. The upper chamber contains the tested biomaterial or bioactive molecules and the lower chamber contains dental cells on a coverslip [67, 68]. This 2D model was adapted to be used in 3D conditions. It consists of the classical in vitro pulp chamber but with cells cultured in a 3D matrix made of polyamide meshes [69].

The most recent innovation in the field of 3D in vitro models to evaluate bioactive molecules is the tooth-on-a-chip device. It is a microphysiologic platform that mimics conditions of the pulp–dentin interface with biomaterials and enables live-cell imaging to study dental pulp-cell response to biomaterials [70]. This microdevice is made by two accessible chambers. One represents the pulp side and the other the cavity with the tested material. The interface reproduces the interface of pulp with dentin-material. The advantage of this miniaturized organ system is that it replicates levels of tissue functionality difficult to achieve with conventional 2D or 3D cell culture models and it also avoids multifactorial challenges that cannot be controlled in vivo [71, 72].

## Ex vivo models for pulp regeneration

By definition, ex vivo models represent a recently extracted entire tissue or organ with minimal alteration from its natural state cultured to preserve vitality under laboratory conditions. These models aim to describe cellular and physiological processes in an environment similar to conditions in a real organ or tissue. Since cytoarchitecture and intercellular connections with ECM are maintained, metabolic processes are closer to the in vivo state than an in vitro model [73].

In the regenerative endodontic field, this kind of laboratory models has been applied to test pulp-capping biomaterials [74, 75]. However, even if these models better mimic clinical situations, they are unsuitable to study regenerative endodontic procedures of necrotic teeth by cell homing through the evaluation of a scaffold inside the root canal. The reason is that to consider a model as ex vivo it should include the whole alive tissue or organ with all the cell types with no or minimal modification. If we translate this to dental pulp regeneration, it means that we should keep intact pulp inside the root. Since dental pulp should be removed to place the scaffold inside the empty canal space, we should not consider root canals with matrices as real ex vivo models. Nevertheless, one study overcame this barrier by using a mandible slice organ culture model to implant a Multidomain Peptide Hydrogel scaffold in the core of intact dental pulp [10]. Ex vivo models for pulp regeneration are presented in Table 3.

**Table 3:** Ex vivo models for dental pulp regeneration

Ex vivo models			
Cell behavior		Scaffold for pulp regeneration	Bioactive molecules (pulp capping)
Normal conditions	Inflammatory conditions	- Mandible slice organ culture model [10]	- Pulp-dentin slice culture system [7, 85–87] - Entire tooth culture system [11, 74, 88]
- Pulp-dentin slice culture system [78] - Pulp-dentin slice Trowel type system [79]	- Pulp-dentin slice culture system [80, 81] - Entire tooth culture system [82, 83]		

### Evaluation of cell behavior in ex vivo models

#### Cell behavior in normal conditions

As mentioned before, studies that use endodontically treated root canal slices could not be considered ex vivo models because pulp tissue and cells are not conserved. Controversially, some studies employ the organ culture system to study the ultrastructure of the odontoblast [76, 77]. With this aim, incisors were extracted and cut longitudinally, and dental pulp was carefully extracted without damaging the odontoblast layer attached to the dentin. Teeth were conserved in culture media and cells were proven to remain alive. This study could be considered as a borderline between in vitro and ex vivo since it did not conserve pulp integrity but a layer of cells corresponding to it.

Pure ex vivo studies have been carried out to find adequate culture conditions to preserve the viability and function of the pulp tissue. This culture method is useful to study the physiological function of odontoblasts and describe dental pulp homeostasis and cellular behavior. With this aim, Hasegawa developed a pulp-dentin slice culture system in rats. He proved that a rocking culture with higher Oxygen levels (95% O2) was more favorable than a hyperbaric stationary culture to maintain cell viability [78].

Later, another study described an ex vivo model based also on the culture of dentin-pulp slices of rat incisors but embedded in an agar-based medium and cultured on floating Millipore filters in Trowel-type cultures. Dental pulp showed no inflammation and preserved vitality for up to 2 weeks [79].

#### Cell behavior after pulp injury-inflammatory conditions

Magliore et al. evaluated pulp tissue response after dentin drilling exposure [80]. They used thick slices of human teeth drilled immediately after extraction and cultured from 3 days to 1 month. They proved that the exposed pulp showed healing aspects such as cell proliferation, neovascularization, and the presence of functional cuboidal cells close to the injured area. Murray et al. presented a model to measure and compare the responses of pulp tissue to cavity preparation and restoration. They examined variables such as the preparation method, remaining dentin thickness, drill speed, conditioning with EDTA, and different filling materials [81]. However, these models do not respect the natural hypoxia present in human dental pulp where oxygen only enters through the apical foramen—a condition that could induce a change in cell metabolism.

Later, Tecles et al. developed an entire tooth culture system to describe the inflammatory reaction and stem cell migration of dental pulp after simulating a clinical exposure [82, 83]. They used entire immature third molars recently extracted to make cavities with or without pulp exposure. Subsequently, the tooth crowns were fixed to the covers of the culture plates, leaving the apical parts floating inside the culture media for up to 4 weeks. This model respects the hypoxia and natural environment of the dental pulp, making it the most advantageous model to evaluate dental pulp response to injury. However, its application has not been translated to pulp revascularization procedures.

Besides, none of these models consider usual conditions in real life as the presence of affected tissue and bacteria that avoid favorable cell response and pulp regeneration. This was proved by an animal model in dogs [84].

### Evaluation of scaffolds for regenerative endodontics

An ex vivo model of mandible slice organ culture was performed to inject in the core of an intact pulp a Multidomain Peptide Hydrogel (MDP) scaffold. Mandibles were dissected, and soft tissues were removed and cut into slices of 2 mm. MDP scaffolds were injected in columns through the entire length of the incisor dental pulp core. Slices were cultured for up to 10 days and histologically evaluated [10]. Results confirmed the biocompatibility of the scaffold and preservation of the surrounding tissue architecture. This model fits into the definition of ex vivo since it keeps all intraoral tissues (bone, periodontal, teeth, and dental pulp). Despite the lack of hypoxia, since the culture is performed in slices of 2 mm instead of along the entire tooth, we consider it the most pertinent model so far for the study of scaffolds for pulp regeneration.

### Evaluation of bioactive molecules and biomaterials for pulp capping

Two types of ex vivo systems related to pulp capping procedures exist the tooth slices system [74], and the entire tooth culture system [8].

Dentin-pulp slices model has been employed to evaluate the responses of human pulp to direct capping with resin adhesive systems, calcium hydroxide, composite resins, or bioceramic types of cement [7, 85, 86]. It has also been used to evaluate pulp reaction to molecules such as Iloprost or VEGF and prove their angiogenic potential by adding them to culture media [7, 87]. Nevertheless, it does not reflect the hypoxia conditions of the human dental pulp.

Facing this disadvantage, the entire tooth culture system has been proposed to evaluate dental pulp response after the application of bioceramic cements in pulp capping procedures [11, 74]. This method also proved pulp healing when collagen or MTA charged with nano plexes of polyethyleneimine and plasmid DNA encoding for FGF-2 and BMP-2 were used for 14 days [88]. This model preserves the natural environment of the dental pulp, making it the closest to reality to evaluating dental pulp response during pulp capping procedures. Ex vivo studies that evaluate the migration potential of bioactive molecules used in cell homing have not been found in the literature.

## In vivo models for pulp regeneration

Animal experiments provide important information about the mechanical behavior of used biomaterials and about their efficacy and biocompatibility [89]. They can be classified into two categories: orthotopic and ectopic models.

### Ectopic models

In ectopic models, the implantation occurs in an abnormal position. These models have been widely used for pulp regeneration due to their low cost and because they are easy to handle (Table 4). Animals that best fit this model are small species such as rodents [90]. Following the objective, we divided these models into two groups. The first group includes models where the scaffolds are implanted subcutaneously in a dorsal zone to evaluate the behavior of dental stem cells and scaffolds. Scaffolds such as poly(lactic-co-glycolic) in rabbits [91] or collagen in mice have been tested [92]. Results demonstrated the organization of newly derived pulp-like tissue.

**Table 4:** Ectopic models used in regenerative dentistry

Cell behaviour (CELL + SCAFFOLD)	Biological response (CARRIER + SCAFFOLD + CELL)		
Scaffold + cell	Tooth slices	Human dental root	
		Cell therapy	Cell Homing
MOUSE - Collagen + DPSC [92] RABIT - DL-Lactid Co glycolide + DPSC [91]	MOUSE - Poly-L-Lactid acid + SHED [93, 98] RAT - Gelatin + PLSC [96] - Gel MA + DPSC, Huvec [97]	MOUSE - Poly-D,L-Lactideglycolide + SCAP,DPSC [100] - Puramatrix + DPSC, PLSC, SHED, BMSC [102] - 3D matrix + SHED, DPSC [101] - Polyethylene glycol, fibrin, colagen + DPSC [102] - PRP + SCAP [103] PIG - Collagen/PLGA + DPSC [104]	MOUSE - GF-laden peptide hydrogel + VEGF, TGF β1, FGF-2 [13] - Plasma rich in GF (PRGF) + TGF β1 + collagen pellet with DPCS in the apex [104]
Pulp-like tissue organization	Osteoblastic, odontoblastic, cementoblastic and fibroblastic differentiation	Pulp-like tissue formation	Pulp-like tissue formation

The second group involves the implantation of more complex systems made by the scaffold placed in a carrier (dentin or root slices) to evaluate the complete biological response. First, studies that implanted tooth slices [93–99]. Results demonstrated osteoblastic, odontoblastic, cementoblastic, and fibroblastic differentiation.

More complex models consist of implanting a Teflon tube or dental root with the tested biomaterial in the canal subcutaneously [13, 100–103]. One end of this graft could be sealed with a bioactive cement, as MTA or Biodentine, or not whereas the other remains open to allow cells and blood to enter. This model has been performed to test cell-free approaches [104] as well as a cell-based therapy for REP proving endodontic space revitalization and pulp-like tissue formation. It was also used to compare the regenerative properties of human stem cells of the apical papilla (SCAPs) seeded in platelet-rich plasma (PRP) as a scaffold [105].

Kodonas et al. performed an ectopic mini-pig model, by implanting inside a post-extraction socket of the jawbone a root fraction with cells and scaffold inside the canal, they prove cell organization and new matrix formation [106].

### Orthotopic models

Orthotopic transplantation is based on the implantation of a graft in its natural location. These models are hazardous due to the necessity to use big animals, rendering them expensive and difficult to manage. However, they have been widely used in the pulp regeneration field because they objectively represent a clinical situation in functional teeth. The method consists in performing a regenerative endodontic treatment in the chosen animal while testing a new technique or material. The protocol for tooth preparation should follow clinical guidelines already established for these procedures. These models are based on the introduction of external cells (cell-based therapy) or cell-free REP (cell homing) (Table 5).

**Table 5:** Orthotropic models for regenerative endodontic treatment of necrotic teeth

Cell-based rep		Rep by cell homing	
Scaffold + cells	Scaffold + cells + bioactive molecules	Classical REP + Photobiomodulation	REP with scaffold
FERRET - Oxidized alginate fibrin + DPSC [16] MINI-PIG - Hydrogel + DPSC [15, 114, 115] DOG - Gelfoam +DPSC [108] - BMDSC/ADSC [96]	DOG - Collagen + DPSC + G-CSF [110]	MOUSE/RAT - Blood clot [90] - Chimeric Mouse: Blood clot + chitosan [107]	DOG – Chitosan [111] – Collagen [112] - Collagen, PRF, blood cloot [84] SHEEP - Collagen + blood clot [17]
Pulp-like tissue formation	Pulp-like tissue formation	Revascularization/ Pulp-like tissue formation	Revascularization/ Pulp-like tissue formation

Many animal species have been standardized to carry out these models. Rodents are not commonly used because they are too small to perform an endodontic treatment on them. However, by using endodontic microscopes, a study evaluated tissue formation after classical REP under photobiomodulation therapy in rats assessing pulp-like tissue formation [90] or in chimeric mice [107].

Dogs are widely used for orthotopic transplantation since their teeth show similar growth patterns and pathophysiology to humans [108]. However, they cannot be considered an ideal model due to differences in the apical region [109]. Nonetheless, several studies testing cell-based therapy that obtained regeneration of pulp-like tissue were performed [14, 96, 110, 111]. For cell homing approach dog models recreating apical periodontitis have been used with blood clots alone or in combination with scaffolds as chitosan hydrogels [111] or collagen [112]. Negative influence in pulp regeneration of important factors were considered by some authors, as advanced age [14] or inflammation due to accidental over instrumentation [84].

In the study mentioned above, non-inflamed samples showed signs of repair, contrary to inflamed canals, proving the importance of controlling periapical inflammation to achieve dental pulp regeneration. This could be explained by the high level of pro-inflammatory macrophages that avoid favorable cell response and pulp regeneration. Although, these cells could be shifted to anti-inflammatory, by reducing bacteria concentration, and enhancing the tissue remodeling process [113].

Therefore, significant attention has been devoted to porcine species due to their similarities with humans. Disadvantages are the high growth rate and excessive weight being difficult to handle.

Cell homing studies obtained and pulp-like tissue formation in mini-pigs [114]. Other studies carried out in a miniature swine model obtained vascularized pulp-like tissue with a layer of dentin-like tissue along the canal walls [15, 115]. However, the disadvantages of using pigs mentioned before remained in this model. Ferrets have also been used to test cell-based pulp regeneration approaches as REP since their teeth anatomy, physiology, histology, and pathology are similar to humans, obtaining pulp-like tissue formation [16, 116].

Sheep have been used for REP by cell homing due to similarities with human dentition, besides being widely available, easy to handle, and cheap compared to other big species [117]. One study examined the response of immature sheep teeth, whose pulp was exposed and infected, and treated within 4 weeks following the classical revitalization protocol with or without collagen as a scaffold. Results assessed root maturation and dentin wall thickening [17].

## Conclusion

### In vitro, ex vivo, and in vivo studies allow a better understanding of regenerative endodontics procedures

Thanks to their development, new cell lines, molecules and matrices are proposed resulting in new approaches to obtain a real pulp regeneration. At in vitro and in ex vivo conditions this regeneration seems to be successful. But in in vivo models the results are closer to clinical conditions and some factors related to the living organism must be taken into consideration.

Pulp capping procedures are in an advanced stage since complex in vitro and ex vivo models close to reality exist allowing representative results that could be easily translated to in vivo evaluation and consequently to clinical practice.

On the other hand, REP of necrotic teeth is insufficiently developed due to the wide utilization of in vivo models that lack reproducibility and predictability because of variability between animals and systemic factors. Future directions include the development of controlled protocols for laboratory models that better simulate REP of necrotic teeth respecting clinical conditions, for example, the presence of inflammatory tissue. Special attention should be paid to the evolution of reproducible ex vivo models that allow a realistic representation of the clinical situation. This would allow us to accurately study different factors such as scaffolds, cells, bioactive molecules, and variations in the clinical situation and protocols to achieve consistent results. Then, *animal* experimentation could be reduced and translation into clinical practice would be faster and safer.
